# Induction of Oxidative Stress on Retinal Pigment Epithelial Cells Triggered a Proangiogenic Environment

**DOI:** 10.3390/ijms27146291

**Published:** 2026-07-15

**Authors:** Mohamed Abdouh, Alicia Goyeneche, Morgan Yurchuk, Miguel N. Burnier

**Affiliations:** 1 Cancer Research Program, Research Institute of the McGill University Health Centre, Montreal, QC H4A 3J1, Canada; alicia.goyeneche@affiliate.mcgill.ca (A.G.); morgan.yurchuk@mail.mcgill.ca (M.Y.); miguel.burnier@mcgill.ca (M.N.B.); 2 The MUHC-McGill University Ocular Pathology & Translational Research Laboratory, McGill University, Montreal, QC H4A 3J1, Canada

**Keywords:** human primary retinal pigment epithelial cells, age-related macular degeneration, disease progression, cell culture, oxidative stress, angiogenesis

## Abstract

Dry age-related macular degeneration (AMD) can progress to wet AMD when leaking capillaries grow under the macula. Although rare, this transition holds significant risk as it causes more rapid and severe vision loss. Oxidative stress is damaging to cellular components and plays a pivotal role in chronic diseases. We aim to determine whether oxidative stress in retinal pigment epithelial (RPE) cells elicits a proangiogenic microenvironment. We exposed human primary RPE cells to hydrogen peroxide (H_2_O_2_), and we analyzed their metabolic activity, and the production of reactive oxygen species (ROS) and angiogenic factors. In addition, we evaluated the potential of RPE cell-conditioned medium (CM) to induce HUVEC cell tube formation. RPE cells exposed to H_2_O_2_ displayed a dose-dependent decrease in their metabolic activity, and increased ROS levels. The analysis of the CM of exposed RPE cells revealed differential expression of a panel of proangiogenic factors. Notably, the expression of the major angiogenic factors (VEGF, FGF) was increased. Exposure of HUVEC cells to the CM of H_2_O_2_-exposed RPE cells promoted tube formation suggestive of microvessel formation. Our findings bring new insights into the role of oxidative stress in altering RPE cell behavior that might have consequences in the progression of AMD towards the exudative form.

## 1. Introduction

Age-related macular degeneration (AMD) is an irreversible and progressive ocular disease, and the leading cause of severe central vision impairment and even permanent blindness among older adults [1,2]. Epidemiological studies have concluded in increased prevalence and burden of AMD across the world, and the number of people suffering from it is expected to reach three billion individuals by 2040 mainly due to the increasing average life expectancy [3,4]. Intrinsic (older age, ethnicity, genetic factors, overweight, hypertension) and environmental factors (cigarette smoking, diet high in saturated fat, extensive chemical and light exposure) are documented risk factors for the disease pathogenesis [1,2,4]. AMD is a major public health problem and is highly associated with a decrease in patients’ quality of life which causes high socio-economic issues [5].

AMD develops following progressive damage to the retinal pigment epithelium (RPE) cells and consequent photoreceptor degeneration at the macula [6]. RPE cells are highly specialized and form a polarized monolayer between the photoreceptor and Bruch’s membrane⁄choriocapillaris. Their dysfunction underlies AMD pathogenesis as they maintain critical functions in the eye posterior segment (i.e., scaffold for photoreceptors, recycling of photoreceptor outer segments, nutrient transport to the neural retina) [7,8].

AMD presents in two forms (dry or wet). Dry AMD is the most prevalent and common form as it affects 80–90% of AMD-affected individuals. Dry AMD occurs when drusen deposits build up in the outer retina beneath the RPE layer with subsequent thinning of the macula and later outer retinal atrophy [2,6]. Dry AMD could progress over time and culminate in neovascularization (growth of immature and leaky blood vessels) associated with retinal exudation (exudative neovascular AMD or wet AMD). Wet AMD is less common (10–20% of AMD cases) but much more serious as it causes 90% of documented blindness [2,6].

Hypoxia, inflammation and oxidative stress are closely linked cellular stress states, and a direct interplay was established between them in a vicious cycle where each one exacerbates the others. Hypoxia increases reactive oxygen species (ROS) production, triggering oxidative stress that contributes to inflammation. These interconnected processes play pivotal roles in chronic diseases, and especially they contribute to AMD pathogenesis [9,10,11].

ROS are produced due to incomplete reduction in molecular oxygen during normal cellular metabolism. At low levels, ROS regulate important physiological processes (i.e., cellular communication, immunity, tissue homeostasis), but when produced at higher levels, they contribute to oxidative stress [12,13,14]. This state contributes to many chronic diseases including AMD where it drives the onset and the progression of the disease [15,16]. Notably, all risk factors of AMD development (i.e., aging, cigarette smoking and light) are inducers of ROS [17,18,19,20]. ROS are unstable and highly reactive with cell components, causing damage to DNA, proteins, and lipids [14]. By dysregulating enzymatic activities and functions of transcription factors, they affect cell growth, differentiation, senescence and viability [21,22].

Oxidative stress is a central contributor to AMD pathogenesis. Indeed, RPE and photoreceptors are particularly susceptible to oxidative damage due to their high metabolic activity, exposure to high oxygen pressure and constant light exposure challenge [9,23,24,25]. Incidentally, ROS hamper RPE cell functioning by inhibiting photoreceptor outer segment recycling, which promotes lipofuscin and drusen accumulation and photoreceptor loss [10,11]. In addition, oxidative stress influences VEGF levels in RPE cells [26]. However, little is known regarding the role of oxidative stress in AMD transition from dry to wet forms.

We undertook this study to determine whether oxidative stress in RPE cells elicits proangiogenic conditions. Using a subtoxic oxidative stress environment, we observed an overexpression of a panel of proangiogenic factors, and subsequent promotion of angiogenesis. This study provides more insights on dry to wet AMD transition and the role of oxidative stress in this process.

## 2. Results

### 2.1. Subtoxic H_2_O_2_ Concentrations Induced Oxidative Stress in RPE with Mild Effects on Cell Vitality

**Figure 1:**
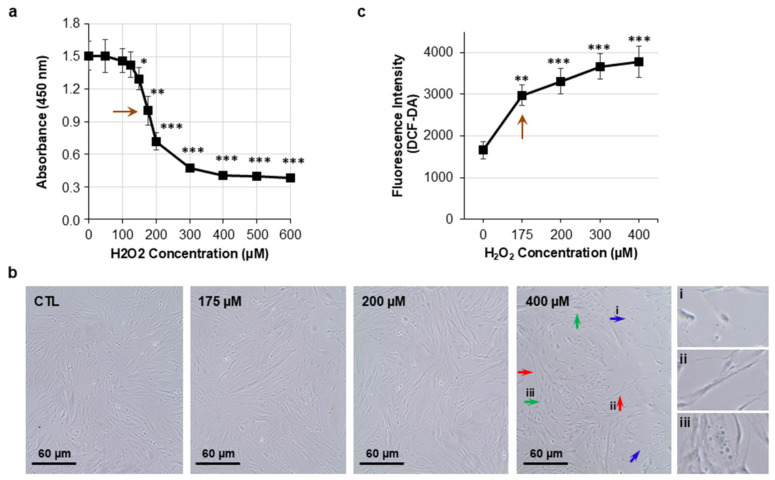
Subtoxic H_2_O_2_ concentrations induced oxidative stress in RPE with mild effects on cell vitality. Human primary RPE cells are exposed to increasing concentrations of H_2_O_2_ (0–600 µM) during 2 h. (**a**) Cells are analyzed for their metabolic activity using the CCK-8 assay. (**b**) Phase contrast views of RPE cells cultured under different exposure conditions. Note that cells cultured under H_2_O_2_ concentrations higher than 200 µM shrink (blue arrowheads; (i), lose adherence (red arrowheads; (ii) and become vacuolized (green arrowheads; (iii)). (**c**) Cells are analyzed for the production of total cellular ROS using the DCF-DA probe. Arrows in (**a**,**c**) designate the subtoxic H_2_O_2_ concentration used in the subsequent assays. Data are presented as mean +/− SD (*n* = 5 independent RPE cell lines each repeated in triplicates, * *p* ˂ 0.05, ** *p* ˂ 0.01, *** *p* ˂ 0.001). Scale bars: 60 µm.

Previous works on oxidative stress induction in RPE cells employed high H_2_O_2_ doses on spontaneously immortalized ARPE19 cells [27,28,29,30,31,32]. In this study, we used human primary RPE cells whose identity was validated throughout the experiment duration by their pigmentation, the expression of RPE markers (KRT8, KRT18 and RPE65) and the absence of melanocyte markers (HMB45). To develop a subtoxic oxidative stress model of RPE cells, we exposed primary RPE cells to increasing concentrations of H_2_O_2_ (0–600 µM) for 2 h (Figure 1). The subtoxic concentration of H_2_O_2_ was determined as the one that achieves oxidative stress (measured by DCF-DA probe) with a mild effect on RPE cell metabolic activity (measured by CCK8 dye) and signs of cell damage (measured by microscope observation). Treatments with H_2_O_2_ concentrations over 200 µM proved too aggressive with catastrophic drop in cell metabolic activity and signs of cell damage (Figure 1a,b). In contrast, RPE cells exposed to H_2_O_2_ at 175 µM developed oxidative stress but with mild effects on cell metabolic activity and no detectable disruption of RPE cell morphology (Figure 1a–c). We kept this concentration as optimal for subsequent analyses.

### 2.2. Proteome from H_2_O_2_-Exposed RPE Cells Clustered Differently from That of Naïve Cells

**Figure 2:**
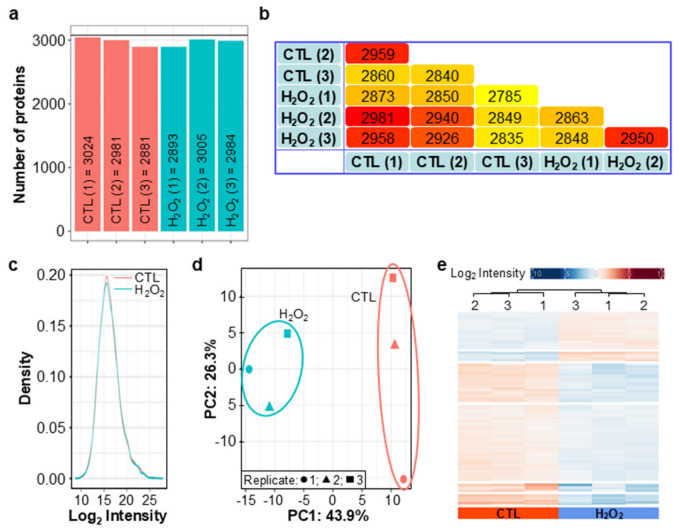
The proteome from H_2_O_2_-exposed RPE cells clusters differently from that of naïve cells. Human primary RPE cells are treated with H_2_O_2_ (175 µM). After overnight incubation, the conditioned medium (CM) is collected and processed for proteomic analysis. (**a**) The graph displays the number of proteins per sample. (**b**) Venn diagram showing the number of proteins shared between the different samples (93–98%). (**c**) Sample correlation assessment. The graph shows the similarities of the protein expression profiles between the analyzed samples. (**d**,**e**) PCA mapping (**d**) and hierarchical heatmap (**e**) show that H_2_O_2_-exposed RPE cells cluster differently from naive cells.

To determine putative factors associated with H_2_O_2_ effects on RPE cells, we performed proteomic analyses. We identified 3052 proteins, of which 93–98% were detected in all analyzed RPE samples (Figure 2a–c). As a readout of the analyzed proteome, we found that most proteins belong to factors secreted in the extracellular space such as growth factors, enzymes, cytokines and binding proteins (Figure S1). Notably, PCA mapping and hierarchical analyses showed that samples clustered differently between H_2_O_2_-exposed and non-exposed cells (Figure 2d,e).

### 2.3. Oxidative Stress Regulated the Expression of Angiogenic Factors

**Figure 3:**
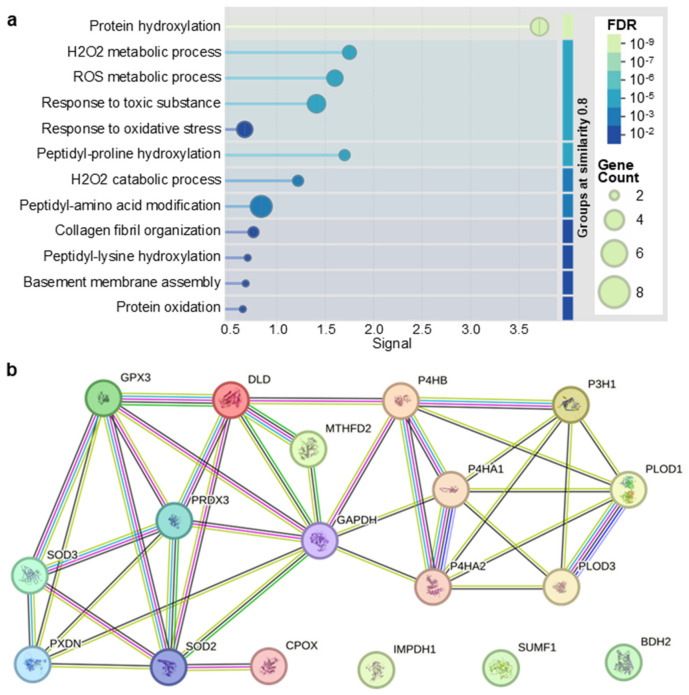
Differentially expressed proteins clustered in pathways related to oxidative stress: (**a**) Gene ontology classification of proteomic dataset for differentially expressed proteins related to oxidative stress in primary RPE cells exposed to H_2_O_2_. Most enriched categories in biological processes are shown. (**b**) Interaction network of proteins identified as associated with oxidative stress highlighting their molecular relationships.

**Figure 4:**
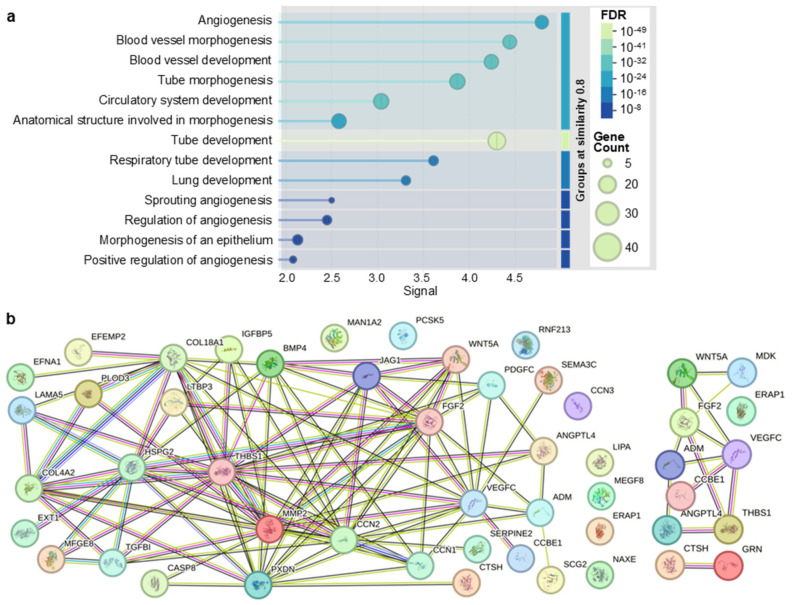
Differentially expressed proteins clustered in pathways related to angiogenesis: (**a**) Gene ontology classification of proteomic dataset for differentially expressed proteins related to angiogenesis (i.e., tube sprouting and vessel growth regulation) in primary RPE cells exposed to H_2_O_2_. Most enriched categories in biological processes are shown. (**b**) Interaction network of proteins identified as associated with angiogenesis highlighting their molecular relationships.

Across conditions, 179 proteins (5.9%) were significantly and differentially expressed between naive and H_2_O_2_-exposed RPE cell-derived conditioned medium (CM). Out of this list, 45 proteins were upregulated and 134 proteins were downregulated in H_2_O_2_-exposed cells (Table S1). To identify physiological processes to which these proteins are related, we clustered and characterized them using STRING gene ontology biological process category (Figure 3 and Figure 4). These analyses highlighted categories consistent with cellular response to oxidative stress and angiogenesis regulators (Tables S1–S3, Figure 3a and Figure 4a). Interestingly, proteins in these categories had molecular relationships as determined by STRING interaction network analyses (Figure 3b and Figure 4b). These data demonstrate that exposure of RPE cells to oxidative stress induces a pro-angiogenic environment.

**Figure 5:**
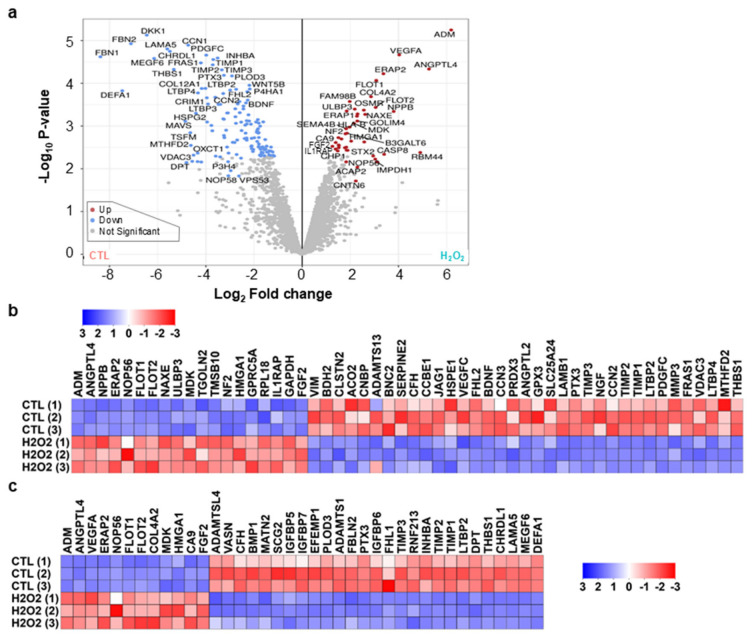
Oxidative stress regulated the expression of angiogenic factors. Human primary RPE cells are treated with H_2_O_2_ (175 µM). After overnight incubation, the conditioned medium (CM) is collected and processed for proteomic analysis. (**a**) Volcano plot representation of 179 proteins significantly and differentially expressed between naive and H_2_O_2_-exposed RPE cells (see Table S1 for the list of the respective proteins) (*t*-test *p*-value < 0.05, Log2 fold change cutoff 1). (**b**,**c**) Heatmap charts depicting the relative expression levels of proteins linked to oxidative stress (**b**) and angiogenesis (**c**) regulation. Column Z score displays relative protein expression levels, where blue and red refer to downexpressed and overexpressed proteins in H_2_O_2_-exposed cells, respectively.

Interestingly, a panel of proteins was either overexpressed or underexpressed in the CM of H_2_O_2_-exposed RPE cells. In relation to response to oxidative stress, we noted that the levels of PRDX3, PLOD1, PLOD3, MTHFD2, GPX3, DLD, HSPE1, PTX3 and THBS1 decreased by at least 60%. In contrast, the levels of IMPDH1, ADM, ANGPTL4, FLOT1, MDK and FGF2 increased by at least two times. Concerning angiogenesis regulators, we found that the levels of SCG2, RNF213 and THBS1 decreased by at least 60%, while those of ADM, ANGPTL4, VEGF and FGF2 increased by at least two times) (Tables S1–S3, Figure 3a, Figure 4a and Figure 5).

To validate these observations, we examined the effect of H_2_O_2_ on RPE cells to release the archetypal angiogenic factor, VEGF (vascular endothelial factor). Using ELISA, we measured VEGF expression in the CM and found significant increase following H_2_O_2_ treatment (Figure S3). Together these data suggest that oxidative stress regulated the expression of angiogenic factors in RPE cells.

### 2.4. Oxidative Stress Promoted Angiogenesis

**Figure 6:**
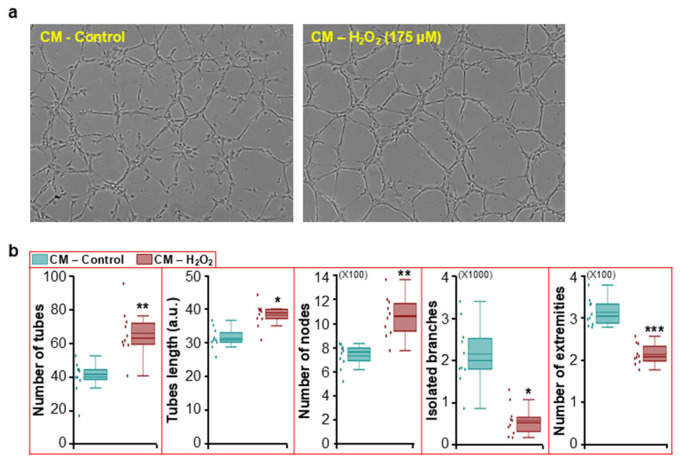
Oxidative stress promoted angiogenesis. Human primary RPE cells are treated with H_2_O_2_ (175 µM). After overnight, the conditioned medium (CM) is collected and added to HUVEC cultures to measure tube formation assay potential. (**a**) Phase contract views of HUVEC cells 4 h following CM addition. (**b**) Metrics of tube networks as calculated in ImageJ software v1.54g with the Angiogenesis Analyzer plugin. Different variables are acquired (i.e., number of tubes; length of tubes, number of nodes, number of isolated branches, number of free extremities). Data are presented as mean ± SD (*n* = CM from 3 independent RPE cell lines, measures acquired in 10 random fields at 20× magnification, * *p* ˂ 0.05, ** *p* ˂ 0.01, *** *p* ˂ 0.001).

As H_2_O_2_ increased the levels of angiogenic factors in RPE cells, we sought to determine the cellular effects of these factors. The treatment of HUVECs with the CM recovered from non-exposed RPE cells gave rise to incomplete tubule formation. In contrast, when we boosted cultures of HUVECs with the CM collected from H_2_O_2_-exposed RPE cells, we realized that this favored the formation of tubular vessel-like structures as judged by the increased number and length of tubes, the increased number of nodes, and the decreased number of isolated branches and free extremities (Figure 6). These data show that the induced oxidative stress in RPE cells affected the secretion of angiogenic factors that elicited a proangiogenic microenvironment auspicious of micro-vessel formation.

## 3. Discussion

**Figure 7:**
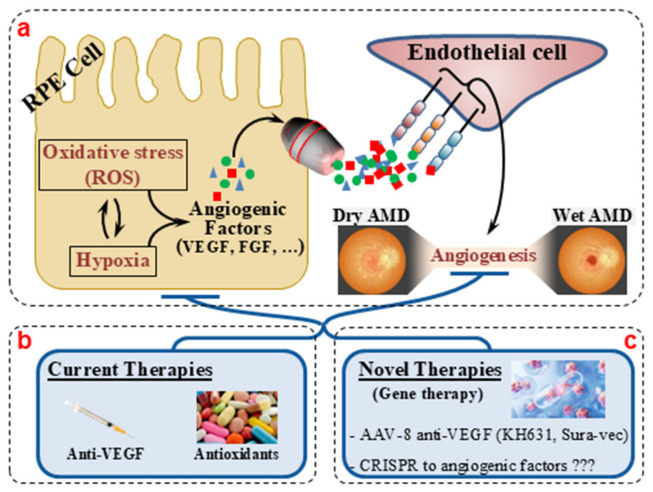
Model summarizing the conclusions of the present study. (**a**) H_2_O_2_-induced oxidative stress in RPE cells caused the secretion of angiogenic factors. These effects elicit a proangiogenic microenvironment suggestive of microvessel formation. (**b**,**c**) This cellular model might serve as a platform for the development of neovascular AMD therapies. (**b**) Antioxidant nutrition supplements can help neutralize ROS and slow oxidative disease progression. (**c**) Antagonizing the release of angiogenic factors may interfere with subsequent neovascular growth. Gene therapy strategies are in progress.

RPE is considered a major target of oxidative stress in the eye and a major source of soluble factors that regulate the microenvironment homeostasis of retina with subsequent implication in the onset and progression of many ocular diseases, such as AMD. A positive regulation exists between oxidative stress and angiogenesis during AMD [9,23,24,25]. H_2_O_2_ is a reliable model of oxidative stress induction in cultured RPE cells. In addition, we used primary human RPE cells from normal eye donors instead of immortalized cell lines. Compared to other studies that used high concentrations of H_2_O_2_ to mimic oxidative stress induction in RPE cells, we employed in this study mild doses of H_2_O_2_ to induce a subtoxic oxidative stress on human primary RPE cells as judged by cell metabolic activity and cell morphology [27,28,29,30,31,32,33]. In summary, we found that under these conditions, RPE cells overexpressed a panel of proangiogenic factors with subsequent promotion of angiogenesis (Figure 7a). This study opens the gate to a better understanding of the role of oxidative stress in the induction of neo-angiogenesis-related ocular disease such as progression of wet AMD. It also offers a platform to test new therapeutic drugs. Many risk factors are related to AMD and interact with each other in its pathogenesis. Notably, oxidative stress and choroidal vascular dysfunction were suggested to be critically involved in AMD pathogenesis [34].

Virtually, all risk factors of AMD development (i.e., aging, cigarette smoking and light) are inducers of oxidative stress with subsequent effects on RPE cell behaviors [17,18,19,20]. In the occurrence, and in line with our present findings, we and others reported that light and cigarette smoke exposure altered angiogenic signaling in human RPE cells [35,36,37,38,39,40]. Aging is associated with drusen-induced hypoxia in the neuroretina, mainly at the macula [41,42,43]. Notably, hypoxia leads to oxidative stress, inflammation, and damage to cellular components, all of which contribute to the underlying pathology of neovascular AMD [11,44]. Specifically, nocturnal hypoxia, often under-diagnosed and common in older demographics, is associated with neovascular AMD, and addressing conditions causing nocturnal hypoxia, such as obstructive sleep apnea, may help manage the risk or progression of the disease [45]. We are reporting in the present study that the archetypal proangiogenic factors, VEGF and FGF-2, were upregulated following oxidative stress. These factors and many other angiogenic factors were found highly expressed in choroidal neovascularization-derived human RPE and aqueous humor of AMD patients [46,47]. These factors have known effects on ocular neovascularization that may underlay neovascular AMD transition [48,49,50,51].

Mitigation therapeutics targeting the oxidative state can help neutralize ROS with potential slowing of the damage. Nutrition supplementation with vitamins C and E, zinc, copper, lutein, zeaxanthin, and phenolic compounds can help mitigate risk and slow disease progression (Figure 7b) [52,53,54,55]. Application of this strategy is promising, as antioxidant-based drugs are routinely used in ophthalmologic clinics. However, the use of antioxidant-rich nutrients is not consistently advocated owing to the related other health issues [56,57]. These systemic complications could potentially be minimized with intravitreal injections for treating AMD [58]. Novel strategies take profit from the role of mitochondria function stimulation to counteract oxidative damage [59,60,61]. On the other hand, as oxidative stress prompted RPE cells to release angiogenic factors, antagonizing these factors may interfere with subsequent neovascular growth and combinatory treatments seem more efficient than solitary factor targeting (Figure 7c) [62,63]. However, these treatments place significant burden on patients as they require frequent repetitive injections [64]. To circumvent this, gene therapies using AAV-8 carrier and targeting angiogenic factors are in progress [65,66,67]. The latter ensures a one-time treatment and guarantees sustained expression for almost 2 years. Strategies using CRISPR technologies are also avenues to target these angiogenic factors [68].

Our findings bring new insights into the involvement of oxidative stress on the alteration of RPE cell behavior and subsequent consequences in the progression of AMD towards the exudative form of the disease. As only about 10% of AMD cases transition to the wet form, one would expect that the exudative form would be the default disease path. Nonetheless, the fact that ROS are associated with dry AMD and only less than 10% of cases transition to the wet form points to the fact that ROS are present during the dry and wet forms, but at different levels. Together, our data provided novel biomarkers of early cellular dysfunction and opportunity for therapeutic intervention in AMD. Our model may be used as a platform for preclinical testing of novel drugs. In our study, we used primary human RPE cells. Due to the hurdles to get access to this type of cell, iPSC-derived RPE cells and retinal organoids hold the potential to model oxidative stress responses and screen novel therapeutics.

## 4. Materials and Methods

### 4.1. Human Primary RPE Cell Culture

Human eyes (*n* = 5, 3 males and 2 females, 55–75 years old) (Table 1) were obtained from the Centre Hospitalier Universitaire de Québec (Centre Universitaire d’Ophtalmologie, Quebec, QC Canada), following informed consent from the donor’s next of kin, and were used in accordance with a protocol approved by the ethic board of the Research Institute of the McGill University Health Centre (RI-MUHC) (IRB #2019–5314) and with the Code of Ethics of the World Medical Association. Primary RPE cells were isolated as we reported previously [69]. Briefly, the whole eye was immersed in 70% ethanol and rinsed in PBS supplemented with 1% penicillin and streptomycin antibiotics (both from Corning, Woodland, CA, USA). The cornea, lens, iris, ciliary body and vitreous humor were removed, and four incisions were made towards the optic nerve to obtain petal-like structure. The retina was gently removed to not disturb the RPE and choroid. The remaining tissue (RPE, choroid and sclera) was immersed into a mixture of collagenases IA and IV (0.5 mg/mL) (Sigma-Aldrich, Oakville, ON, Canada) for 1 h at 37 °C to loosen the RPE layer from the choroid. The RPE layer was gently scraped off. Supernatant was collected and the RPE layer fragments were triturated by repeated pipetting with gradual pipette sizes. Cell suspension was filtered through a 40 µm cell strainer. RPE cells were recovered in DMEM-F12 medium supplemented with 10% fetal bovine serum (FBS) and 1% penicillin–streptomycin antibiotics (all from Corning, Woodland, CA, USA). Three to four days later, the medium was changed and replenished every three days afterwards. At 80% confluency, cells were passaged using trypsin/EDTA (Corning, Woodland, CA, USA) for 2–3 min at 37 °C.

The identity of the isolated RPE cells was verified by analyzing the expression of cytoplasmic pigmentation, the expression of RPE cell markers (KRT8, KRT18, RPE65) and the absence of melanocyte marker (HMB-45) [69].

For oxidative stress induction, culture medium was removed and cells were washed with PBS. RPE cells were treated with increasing amounts of H_2_O_2_ (Sigma-Aldrich, Oakville, ON, Canada) in FBS-free medium for 2 h. The optimal H_2_O_2_ concentration was chosen as the one that achieved subtoxic oxidative stress without catastrophic loss of vitality (as measured by the CCK-8 and DCFDA assays).

Human umbilical vein endothelial cells (HUVEC, PCS-100-010) were grown in Vascular Cell Basal Medium (PCS-100-030) supplemented with Endothelial Cell Growth Kit-BBE (PCS-100-040, ATCC) as recommended by the manufacturer (Cedarlane, Burlington, ON, Canada). All experiments were performed at cell passages between 2 and 5.

### 4.2. RPE Cell Vitality Analyses

For the assessment of the metabolic activity, cells were plated in transparent 96-well plates (ThermoFisher, Saint-Laurent, QC, Canada). After H_2_O_2_ treatment, medium was removed and cells were washed with excess PBS. CCK8 (10%; Dojindo Molecular Technologies, Burlington, ON, Canada) was added to cells. Two hours later, the absorbance was read at 450 nm using an Infinite M200Pro microplate reader (Tecan, Morgan Hill, CA, USA). In parallel, pictures of cell culture were scanned using an inverted photonic microscope.

### 4.3. Reactive Oxygen Species (ROS) Level Detection

Cells were plated in black clear-bottom 96-well plates (Costar, Kennebunk, ME, USA). After H_2_O_2_ treatment, medium was removed and cells were washed with excess PBS. ROS levels were determined using the 2′, 7′-dichlorodihydroluorescein diacetate (DCF-DA) probe according to the manufacturer protocol (abcam, Waltham, MA, USA). Briefly, DCF-DA probe was added at 10 μM to cells for 30 min at 37 °C. After additional washes with PBS, the fluorescence was quantified at excitation 485 nm and emission 530 nm, using an Infinite M200Pro microplate reader (Tecan). Reading parameters were introduced manually to normalize for fluorescence measurements between experiments. To this purpose, reading mode was set to Fluorescence Top Reading, and the gain and Z-position were set manually to 100 and 14,600 µm, respectively. Also, the number of flashes and the integration time were set at 25 and 20 µs, respectively.

### 4.4. Preparation of Conditioned Medium of H_2_O_2_-Exposed RPE Cells and Analytical Assays

RPE cells were exposed to H_2_O_2_ (175 µM) for 24 h, and the conditioned medium (CM) was collected and centrifuged at 300 g for 5 min at 4 °C to pellet floating cells and debris. The CM was aliquoted and stored at −80 °C for use in subsequent analytical assays (i.e., levels of produced soluble angiogenic factors and effects on HUVEC cell-mediated tube formation).

#### 4.4.1. Proteomic Mass Spectrometry Analysis

CM (1 mL) was thawed on ice and processed for UPLC/MS-MS spectrometry at the Proteomics and Molecular Analysis Platform of the RI-MUHC using an Orbitrap Astral Mass Spectrometer (ThermoFisher Scientific, Waltham, MA, USA). Samples were cleared at 500 g for 5 min at 4 °C, then filtered through a 0.22 µm filter. Filtrates were precipitated by adding sodium deoxycholate (0.1%) and trichloroacetic acid (10%). Following overnight incubation at −20 °C, proteins were pelleted at 10,000 g for 15 min at 4 °C. Pellets were recovered in 6 M urea. For each sample, proteins were loaded onto a single stacking gel band to remove lipids, detergents and salts. The gel band was reduced with DTT, alkylated with iodoacetic acid and digested with trypsin. Extracted peptides were re-solubilized in 0.1% aqueous formic acid.

LC–MS/MS analysis was performed on a Thermo FAIMS Duo Pro/Orbitrap Astral MS system coupled to a Thermo Scientific Vanquish Neo UHPLC using mobile phase A: 0.1% formic acid in water and mobile phase B: 0.1% formic acid/80% acetonitrile in water. Briefly, 200 ng of peptide/sample were loaded onto a Thermo PEPMAP Neo 300 µM × 5 mM trap column into an IonOpticks Aurora Rapid 150 µM × 8 cm separation column heated to 50 °C using a 180 SPD gradient (4–40% mobile phase B over 7 min + wash + reconditioning steps). On the Orbitrap Astral MS, a FAIMS voltage of −45 V (to emphasize 2+ *m*/*z*) was used. MS1 spectra were recorded using the Orbitrap analyzer at a resolution of 240,000 from *m*/*z* 380 to 980 using an automated gate control (AGC) target of 500% and a maximum injection time of 3 ms. For MS2 in nDIA mode using the Astral analyzer, 200 nonoverlapping isolation windows from *m*/*z* 380 to 980 of 3 *m*/*z* were used. HCD collision energy was set to 25% and the RF lens at 40%.

DIA data were searched against Human Uniprot 2025 sequences using Fragpipe v23.1 using the DIA-Speclib-Quant workflow using all nDIA files to create the library with quantification using DIA-NN v1.8.2beta8 functions in Fragpipe. Fragpipe modules used in the method are: MSFragger [70,71]; MSBooster [72]; Percolator [73]; ProteinProphet [74]; Philosopher [75]; Spectral library generation [76]; DIA quantification with DIA-NN [77]. Data were visualized and plotted using DIA-Analyst v0.10.5. (https://analyst-suites.org/apps/dia-analyst/; Monash Proteomics & Metabolomics Platform (accessed on 15 April 2026)).

A *p*-value cut-off of 0.05 and a fold-value change of ≥2 were used to identify the differentially expressed proteins. Protein lists were uploaded into the DAVID or STRING Bioinformatics database for the analysis of functional gene enrichment and annotation (gene ontology analyses) and protein network association. In addition, bioinformatic analyses were performed using the FunRich software (version 3.1.3) for heatmap construction.

#### 4.4.2. Enzyme-Linked Immunosorbent Assay (ELISA)

To validate the levels of the vascular endothelial growth factor (VEGF) levels, we used the Human VEGF Quantikine ELISA kit (R&D Systems, DVE00, Minneapolis, MN, USA) per the manufacturer’s instructions. Briefly, CM was thawed on ice and concentrated using Amicon Ultra 4 Centrifugal Filters with a 3 kDa molecular weight cutoff (SIGMA). The eluates were added to the pre-coated wells in duplicate and incubated for 2 h at room temperature (RT). After washing, HRP-conjugated anti-VEGF was added to the wells and incubated for 2 h at RT. After signal developing, the optical density at 450 nm was determined using a TECAN microplate reader. Second readings were done at 540 nm and subtracted from readings at 450 nm to correct for optical imperfections in the plate. Obtained values were normalized with respect to the cell number per well. Standard concentrations of recombinant VEGF were used in parallel to produce a standard curve to calculate VEGF concentrations (pg/mL) in the CM.

#### 4.4.3. In Vitro Tube Formation Assay

Plates (24-well) were coated with Matrigel Growth Factor Reduced Basement Membrane Matrix (Corning). HUVEC cells (5 × 10^4^) were plated per well and cultured covered by 0.2 mL CM. Cultures were incubated for 4 h and ten random fields per well were photographed under a phase-contrast microscope. For tube network quantification, images were opened in ImageJ software (version v1.54g) with the Angiogenesis Analyzer plugin [78]. Different variables were acquired (i.e., number of tubes; length of tubes, number of nodes, number of isolated branches, number of free extremities).

### 4.5. Statistical Analyses

All experiments were performed with 5 independent primary RPE cell cultures except for the proteomic analyses, in which we used only 3 RPE lines. Data were compared using an ANOVA followed by the Dunnett post hoc test for multiple comparisons with one control group. A *p*-value < 0.05 was considered statistically significant.

## Figures and Tables

**Table 1:** RPE cell donors’ information.

Donor’s ID	Sex, Age	Maximum Culture Length
RPE0829X	Female, 73 y	58 days
RPE0708X	Female, 68 y	75 days
RPE0712Y	Male, 77 y	70 days
RPE0924Y	Male, 80 y	70 days
RPE1005Y	Male, 71 y	55 days

## Data Availability

The data presented in this study are available on request from the corresponding author due to formal request.

## Supplementary Materials

The following supporting information can be downloaded at: https://www.mdpi.com/article/10.3390/ijms27146291/s1.

## Abbreviations

The following abbreviations are used in this manuscript:

AMD	Age-Related Macular Degeneration
CCK8	Cell Counting Kit 8
CM	Conditioned Medium
DCF-DA	Dichloro-Dihydro-Fluorescein DiAcetate
FBS	Fetal Bovine Serum
FGF2	Basic Fibroblast Growth Factor
H_2_O_2_	Hydrogen Peroxide
HUVECs	Human Umbilical Vein Endothelial Cells
IRB	Institutional Review Board
PBM	PhotoBioModulation
PCA	Principal Component Analysis
RI-MUHC	Research Institute of the McGill University Health Centre
ROS	Reactive Oxygen Species
RPE	Retinal Pigment Epithelial
SD	Standard Deviation
UPLC/MS-MS	Ultra-Performance Liquid Chromatography/Tandem Mass Spectrometry
VEGF	Vascular Endothelium Growth Factor
ELISA	Enzyme-Linked Immunosorbent Assay
