# Supplementary material for: Induction of Oxidative Stress on Retinal Pigment Epithelial Cells Triggered a Proangiogenic Environment
Source: Int J Mol Sci. 2026 Jul 15;27(14):6291. doi: 10.3390/ijms27146291 (PMC13409842; doi:10.3390/ijms27146291)
Supplement: Supplementary file 1 [file ijms-27-06291-s001.zip › ijms-4398126-supplementary.pdf]

Induction of oxidative stress on retinal pigment epithelial cells triggered a proangiogenic environment

Supplementary Files:

a

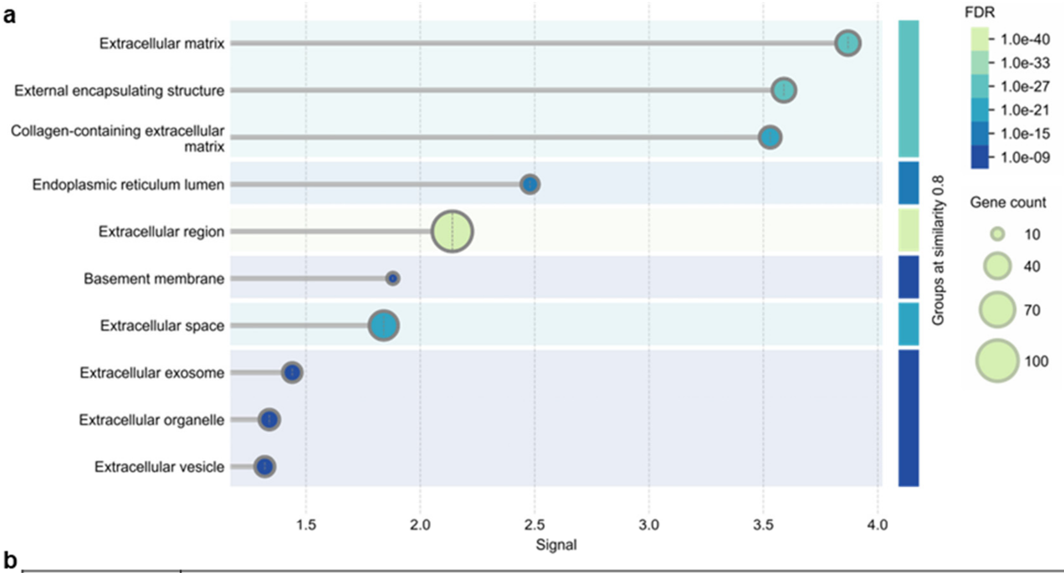

b

Functions	Protein ID
Growth factor	GRN, TIPM1, BMP4, FGF2, WNT5A, MFGE8, ANGPTL4, NGF, ANGPTL2, BDNF, VEGF.
Binding protein	FSTL3, IGFBP5, THBS1, VWF, LTBP2, NID1, IGFBP7, IGFBP6, FLOT1, EFEMP1, FLOT2, LTBP1, CCBE1.
Enzyme	PLOD1, PLOD3, CTSC, MMP2, CTSH, PXDN, ENPP2, TIMP2, TIMP3, ADAMTS1, ADAMTS3, NCSTN, ERAP1, MMP3.
Cytokine	CCN3, ANOS1, MDK, IL6, CCN1, TGFB1.

Supplementary Figure S1. Subcellular localization of analyzed proteome. (a) Cluster enrichment. **(b)** Example of proteins enriched in the analyzed proteome.

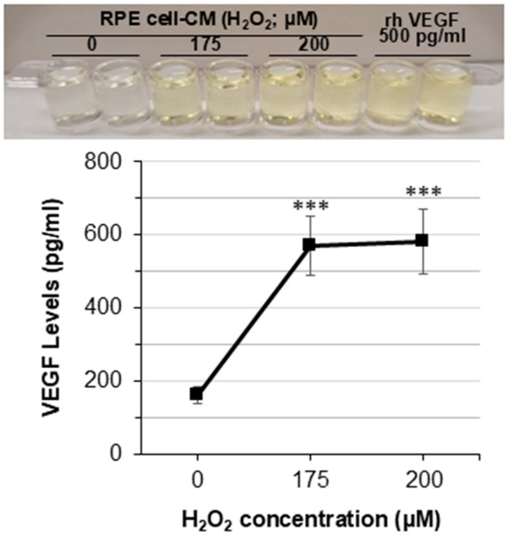

Supplementary Figure S2. Human primary RPE cells were treated with H_2O_2 (175 or 200 µM). After overnight, the conditioned medium (CM) was collected and processed for anti-VEGF ELISA. Data are presented as mean ± SD (*n* = 3 independent RPE cell lines, each repeated in duplicate, *** P < 0.05).

Supplementary Table S1. List of proteins differentially expressed in H2O2-exposed cells and naive cells

Protein ID	CTL 1	CTL 2	CTL 3	H2O2 1	H2O2 2	H2O2 3	H2O2_vs _CTL	Fold change	P value
ADM	13,74	14,19	13,49	17,04	16,67	17,00	3,09	8,54	0,00001
ANGPTL4	13,28	13,18	14,10	16,28	16,08	16,10	2,63	6,20	0,00005
RBM44	16,05	NA	NA	18,75	18,26	NA	2,45	5,47	0,00416
VEGFA	17,41	17,76	17,53	19,83	19,52	19,39	2,02	4,04	0,00002
NPPB	20,86	21,61	21,76	23,61	23,03	23,28	1,90	3,73	0,00046
CASP8	9,65	NA	9,49	10,90	11,64	NA	1,70	3,25	0,00459
ERAP2	19,77	20,13	20,06	21,67	21,47	21,88	1,69	3,22	0,00006
IMPDH1	11,82	10,98	NA	13,02	12,51	13,37	1,56	2,95	0,00700
FLOT1	16,10	16,35	16,48	17,84	17,68	18,03	1,54	2,90	0,00009
FLOT2	16,96	17,38	17,57	18,70	18,67	19,12	1,53	2,88	0,00037
NOP56	12,25	12,44	NA	13,38	14,45	13,77	1,52	2,86	0,00595
STX2	11,49	10,91	NA	NA	12,78	12,57	1,47	2,78	0,00502
COL4A2	21,81	22,07	22,11	23,27	23,29	23,72	1,43	2,69	0,00021
NAXE	14,70	14,62	NA	16,14	15,87	15,89	1,31	2,48	0,00053
HMGN1	14,84	14,60	14,26	15,98	16,20	15,40	1,29	2,44	0,00236
OSMR	16,49	16,76	17,03	18,18	17,87	18,08	1,28	2,43	0,00042
NCSTN	18,52	18,78	18,69	19,81	19,58	20,05	1,15	2,22	0,00052
ACAP2	12,14	11,74	NA	NA	13,24	12,92	1,15	2,21	0,00915
GOLIM4	17,32	17,52	17,58	18,67	18,33	18,87	1,14	2,21	0,00077
ERAP1	20,93	21,18	21,30	22,33	22,04	22,44	1,14	2,20	0,00059
CNTN6	NA	11,79	11,77	NA	NA	12,90	1,11	2,16	0,01928
ULBP3	13,75	14,13	13,79	15,04	14,94	14,97	1,09	2,13	0,00040
MDK	12,31	12,34	11,98	13,08	13,48	13,30	1,07	2,11	0,00086
MAN1A2	13,25	13,33	13,00	14,51	13,89	14,25	1,02	2,02	0,00226
FAM98B	14,55	14,55	14,52	15,63	15,43	15,52	0,99	1,98	0,00027
HLA-B	16,67	17,02	17,05	17,93	17,73	18,03	0,99	1,98	0,00106
TGOLN2	17,91	18,40	18,40	19,32	18,90	19,32	0,94	1,92	0,00370
B3GALT6	15,51	15,46	15,79	16,51	16,33	16,71	0,93	1,90	0,00147
TMSB10	17,56	17,53	17,70	18,62	18,46	18,49	0,92	1,90	0,00045
SUMF1	17,61	18,30	18,03	18,89	18,83	18,99	0,92	1,90	0,00368
CBX5	15,17	14,66	14,59	15,73	15,82	15,62	0,92	1,89	0,00312
CHP1	12,38	12,38	12,45	NA	13,05	13,58	0,91	1,88	0,00689
SEMA4B	18,15	18,53	18,35	19,17	19,20	19,39	0,91	1,88	0,00115
NF2	14,93	14,60	14,54	15,69	15,56	15,55	0,91	1,88	0,00115
HMGA1	17,42	17,14	17,09	18,04	18,31	18,01	0,90	1,86	0,00152
GPRC5A	18,07	18,35	18,60	19,12	19,14	19,43	0,89	1,85	0,00322
NPTXR	17,33	17,59	17,53	18,31	18,12	18,46	0,82	1,76	0,00201
MPZL1	16,76	16,94	16,73	17,64	17,36	17,72	0,76	1,70	0,00287
MAN1B1	16,87	17,08	16,81	17,72	17,48	17,84	0,76	1,69	0,00322
RPL18	15,88	15,48	15,74	16,53	16,39	16,45	0,76	1,69	0,00278
CA9	18,69	18,88	18,84	19,56	19,42	19,68	0,75	1,68	0,00187
IL1RAP	17,64	18,06	17,75	18,54	18,47	18,65	0,74	1,67	0,00383
GAPDH	23,00	23,19	23,20	23,91	23,84	23,73	0,70	1,62	0,00244
RAN	18,69	18,56	18,65	19,29	19,51	19,17	0,69	1,62	0,00346
FGF2	14,65	14,73	14,63	15,21	15,37	15,31	0,63	1,55	0,00299
KRT18	19,78	19,77	19,78	19,30	19,07	19,22	-0,58	0,67	0,00504

KRT7	18,49	18,32	18,24	17,78	17,72	17,76	-0,60	0,66	0,00483
DNAJC3	16,84	16,68	16,69	16,15	16,02	16,19	-0,61	0,65	0,00405
VIM	23,15	23,39	23,19	22,68	22,50	22,61	-0,65	0,64	0,00386
BDH2	15,87	15,87	15,68	15,15	15,22	15,11	-0,65	0,64	0,00311
CLSTN2	20,60	20,84	20,75	20,12	19,90	20,19	-0,66	0,63	0,00486
P3H1	16,90	17,06	16,87	16,11	16,24	16,43	-0,68	0,62	0,00379
FUT8	16,62	16,75	16,62	16,20	15,91	15,82	-0,69	0,62	0,00471
IL6	16,06	15,83	15,66	15,07	15,18	15,20	-0,70	0,61	0,00401
P4HB	20,17	19,93	19,82	19,39	19,27	19,13	-0,71	0,61	0,00406
ACO2	15,43	15,00	15,19	14,52	14,55	14,36	-0,73	0,60	0,00408
CFI	15,68	15,90	16,03	15,02	15,19	15,20	-0,74	0,60	0,00295
LIPA	18,13	18,48	18,23	17,36	17,67	17,54	-0,76	0,59	0,00346
TGFBI	24,32	24,57	24,37	23,68	23,51	23,77	-0,77	0,59	0,00208
ATP6AP1	16,88	17,12	17,26	16,15	16,26	16,50	-0,78	0,58	0,00361
MMP2	21,94	22,44	22,37	21,51	21,33	21,56	-0,79	0,58	0,00493
CNBP	16,02	15,62	15,98	14,99	14,99	15,23	-0,80	0,57	0,00327
COLGALT1	16,34	16,32	16,17	15,50	15,46	15,45	-0,81	0,57	0,00080
ADAMTSL4	19,02	19,20	19,04	18,06	18,16	18,59	-0,82	0,57	0,00422
BNC2	17,64	17,75	18,07	17,17	16,84	16,99	-0,82	0,57	0,00324
GRN	18,59	19,12	18,87	18,00	18,00	18,10	-0,82	0,57	0,00306
SERPINE2	16,49	16,80	16,39	15,52	15,78	15,87	-0,84	0,56	0,00322
CTSO	17,14	17,72	17,39	16,46	16,57	16,69	-0,84	0,56	0,00407
GNS	21,13	21,50	21,51	20,54	20,26	20,77	-0,85	0,55	0,00456
BOC	15,14	15,06	15,47	14,58	14,05	14,46	-0,86	0,55	0,00504
EXT2	19,38	19,96	19,81	18,88	18,77	18,91	-0,86	0,55	0,00349
EXT1	19,47	19,74	19,52	18,67	18,62	18,82	-0,87	0,55	0,00101
CSRP2	16,86	17,32	17,16	15,93	16,42	16,37	-0,88	0,54	0,00481
SGSH	17,84	18,03	17,82	16,78	16,91	17,31	-0,90	0,54	0,00262
P4HA2	17,26	17,14	17,11	16,48	16,34	15,99	-0,90	0,54	0,00186
CFH	15,71	16,11	16,10	14,93	14,96	15,33	-0,90	0,54	0,00301
LPP	16,66	16,77	16,78	15,64	15,94	15,92	-0,90	0,53	0,00085
VASN	20,17	20,39	20,11	19,21	19,21	19,54	-0,90	0,53	0,00143
SLIT3	17,79	18,06	17,97	16,87	16,86	17,37	-0,90	0,53	0,00310
SSBP1	16,57	16,30	16,31	15,70	15,43	15,32	-0,91	0,53	0,00146
CANT1	18,18	18,58	18,32	17,39	17,39	17,55	-0,92	0,53	0,00116
MFGE8	19,51	19,74	19,72	18,71	18,63	18,87	-0,92	0,53	0,00071
CCBE1	21,03	21,28	21,41	20,31	20,06	20,54	-0,93	0,52	0,00229
BMP1	19,53	19,96	19,77	18,81	18,70	18,94	-0,93	0,52	0,00126
ENPP2	21,23	21,64	21,38	20,41	20,35	20,65	-0,94	0,52	0,00139
JAG1	14,64	14,91	14,83	14,02	13,77	13,72	-0,96	0,52	0,00081
MATN2	22,28	22,57	22,53	21,23	21,39	21,77	-0,99	0,50	0,00189
HSPE1	18,31	18,01	17,78	17,30	17,06	16,70	-1,02	0,49	0,00352
WNT5A	15,16	14,86	15,65	14,05	14,35	14,21	-1,02	0,49	0,00394
EFNB1	15,49	15,94	15,85	NA	14,63	14,80	-1,05	0,48	0,00340
TRIP6	15,37	15,59	15,72	14,26	14,42	14,86	-1,05	0,48	0,00194
FAM98A	14,02	13,73	13,62	13,05	12,79	12,37	-1,05	0,48	0,00291
VEGFC	17,21	17,59	17,25	15,89	16,44	16,55	-1,06	0,48	0,00303
SCG2	14,81	15,35	15,03	14,08	13,91	14,00	-1,06	0,48	0,00090
P4HA1	17,66	17,72	17,68	16,54	16,69	16,57	-1,09	0,47	0,00014
WNT5B	14,49	14,48	14,46	13,44	13,33	13,38	-1,10	0,47	0,00011

CTSH	22,85	23,25	23,20	21,97	21,83	22,19	-1,10	0,47	0,00076
NUCB2	18,03	18,44	18,26	17,29	16,97	17,13	-1,12	0,46	0,00055
BDNF	15,98	16,32	16,30	15,05	15,07	15,08	-1,13	0,46	0,00025
FHL2	16,64	16,56	16,54	15,29	15,51	15,52	-1,14	0,45	0,00016
MATN3	16,86	17,07	16,79	15,45	15,79	16,03	-1,15	0,45	0,00089
OLFML2A	13,52	13,55	NA	12,54	12,52	12,10	-1,15	0,45	0,00214
SEMA3C	19,42	19,71	19,51	18,19	18,30	18,70	-1,15	0,45	0,00075
CCN3	19,44	20,09	20,10	18,81	18,54	18,82	-1,15	0,45	0,00191
SPESP1	15,54	15,65	15,80	14,47	14,33	14,69	-1,17	0,45	0,00029
NID1	19,59	19,97	19,99	18,42	18,57	19,04	-1,18	0,44	0,00147
CTSC	20,29	20,55	20,58	19,05	19,19	19,64	-1,18	0,44	0,00095
HTRA1	18,02	18,55	18,29	16,87	16,92	17,49	-1,19	0,44	0,00196
PPIC	18,13	18,63	18,52	17,23	17,19	17,20	-1,22	0,43	0,00034
PCSK5	16,44	16,74	16,70	15,18	15,41	15,63	-1,22	0,43	0,00040
IGFBP5	20,82	21,46	21,15	20,15	19,88	19,62	-1,26	0,42	0,00128
IGFBP7	23,38	24,02	23,98	22,62	22,42	22,54	-1,27	0,42	0,00081
VWF	15,02	15,56	15,58	NA	13,83	14,41	-1,27	0,41	0,00565
ANOS1	16,70	16,90	16,69	15,32	15,25	15,82	-1,30	0,41	0,00049
PLOD1	20,72	21,16	21,06	19,63	19,56	19,85	-1,30	0,41	0,00027
VPS53	NA	12,21	12,44	NA	11,01	NA	-1,31	0,40	0,01475
EFEMP1	22,40	22,92	22,71	21,38	21,17	21,47	-1,34	0,40	0,00031
FSTL3	18,38	19,38	18,90	17,64	17,36	17,61	-1,35	0,39	0,00212
PRDX3	16,90	16,38	16,86	15,96	14,83	15,27	-1,36	0,39	0,00464
ANGPTL2	19,30	19,55	19,18	17,86	17,91	18,14	-1,37	0,39	0,00014
UBE2J1	13,08	12,98	12,82	NA	11,38	11,79	-1,38	0,39	0,00087
GPX3	18,19	18,95	18,48	17,15	17,24	17,06	-1,39	0,38	0,00061
EFEMP2	20,40	20,95	20,82	19,30	19,16	19,55	-1,39	0,38	0,00040
FLYWCH2	13,43	13,39	NA	NA	11,83	12,18	-1,40	0,38	0,00242
SEC11A	13,27	NA	NA	11,75	11,93	11,81	-1,44	0,37	0,00241
PLOD3	19,09	19,41	19,20	17,80	17,64	17,90	-1,46	0,36	0,00007
P3H4	14,24	13,78	NA	12,89	12,16	NA	-1,48	0,36	0,01106
ADAMTS1	16,88	17,28	17,31	15,48	15,67	15,83	-1,50	0,35	0,00014
LTBP1	14,91	15,01	14,76	12,63	13,42	14,04	-1,53	0,35	0,00429
NOP58	11,90	12,32	NA	NA	10,58	NA	-1,53	0,35	0,01458
SLC25A24	13,75	13,10	NA	12,44	11,55	11,67	-1,54	0,34	0,00684
ADAMTS3	15,21	15,52	14,81	13,63	13,39	13,90	-1,54	0,34	0,00049
DLD	17,02	16,22	16,33	14,88	14,96	15,06	-1,56	0,34	0,00050
BMP4	14,65	14,94	14,15	NA	13,28	12,76	-1,56	0,34	0,00310
FBLN2	16,61	17,00	16,96	14,82	15,18	15,84	-1,58	0,33	0,00125
FHL1	15,35	15,59	16,42	13,83	14,32	14,36	-1,62	0,33	0,00187
ATP5F1D	16,11	15,58	15,93	14,87	13,79	14,08	-1,62	0,32	0,00166
LAMB1	20,47	20,82	20,95	18,90	18,83	19,63	-1,63	0,32	0,00070
PTX3	17,78	17,82	17,86	16,00	16,09	16,49	-1,63	0,32	0,00006
EFNA1	17,21	17,60	17,49	15,69	15,39	16,31	-1,64	0,32	0,00068
IGFBP6	21,33	21,86	21,85	20,04	19,84	20,24	-1,64	0,32	0,00017
TIMP3	17,14	17,62	17,48	15,68	15,77	15,78	-1,67	0,31	0,00005
NGF	16,13	16,45	15,74	14,17	14,35	14,70	-1,70	0,31	0,00031
PAM16	14,68	NA	NA	NA	12,98	12,98	-1,70	0,31	0,00526
RNF213	14,59	14,62	14,79	12,90	13,39	12,50	-1,74	0,30	0,00031
INHBA	18,12	18,35	17,93	16,47	16,33	16,35	-1,76	0,30	0,00003

MEGF8	16,95	17,26	16,89	14,34	15,29	16,08	-1,80	0,29	0,00506
CCN2	22,10	22,74	22,91	20,92	20,71	20,71	-1,80	0,29	0,00021
TIMP1	20,34	20,81	20,73	18,71	18,80	18,83	-1,85	0,28	0,00003
TIMP2	22,38	22,79	22,64	20,52	20,84	20,88	-1,86	0,28	0,00004
HS3ST1	15,42	15,46	15,43	12,99	13,39	14,33	-1,87	0,27	0,00129
TACC2	15,35	15,66	14,83	12,85	13,49	13,85	-1,89	0,27	0,00097
LTBP3	20,96	21,31	21,21	18,75	19,12	19,74	-1,96	0,26	0,00031
PTCD3	12,61	12,64	12,41	10,58	NA	NA	-1,97	0,25	0,00080
CRIM1	19,72	20,20	20,21	17,62	18,11	18,46	-1,98	0,25	0,00022
PDGFC	18,20	18,64	18,56	16,58	16,55	16,30	-1,99	0,25	0,00002
LTBP2	21,66	22,04	21,77	19,47	19,70	20,28	-2,01	0,25	0,00013
COL12A1	22,65	22,94	23,05	20,85	20,34	21,20	-2,09	0,24	0,00013
ITGBL1	14,35	15,50	15,63	13,52	NA	12,61	-2,10	0,23	0,00695
FRAS1	17,53	17,76	17,66	15,53	15,23	15,88	-2,10	0,23	0,00003
LTBP4	18,94	19,29	19,09	16,47	16,89	17,47	-2,16	0,22	0,00017
OXCT1	17,03	16,60	16,72	15,81	14,02	14,01	-2,17	0,22	0,00430
MMP3	15,07	15,01	14,37	11,87	13,42	NA	-2,17	0,22	0,00680
HSPG2	22,55	22,94	22,88	20,10	20,33	21,33	-2,21	0,22	0,00050
VDAC3	16,14	15,61	16,17	14,48	12,95	NA	-2,26	0,21	0,00480
ATP5F1C	15,89	NA	NA	14,09	13,03	13,70	-2,28	0,21	0,00670
MTHFD2	16,00	15,15	15,17	13,69	12,56	NA	-2,31	0,20	0,00284
TSFM	15,92	15,33	15,52	13,27	NA	NA	-2,32	0,20	0,00143
CCN1	21,97	22,32	22,21	19,56	19,74	20,09	-2,37	0,19	0,00001
DPT	NA	NA	14,46	12,49	11,40	12,27	-2,40	0,19	0,00713
MAVS	13,12	12,90	13,40	NA	10,71	NA	-2,43	0,19	0,00077
THBS1	25,28	25,69	25,79	22,54	22,80	23,42	-2,67	0,16	0,00005
CHRDL1	17,70	18,09	17,81	14,68	15,23	15,44	-2,75	0,15	0,00002
LAMA5	18,69	18,92	18,86	15,66	15,91	16,49	-2,80	0,14	0,00002
MEGF6	17,60	18,00	17,94	14,17	15,06	15,08	-3,07	0,12	0,00003
DKK1	17,38	17,79	17,73	13,95	14,67	14,60	-3,23	0,11	0,00001
FBN2	19,14	19,72	19,65	16,11	15,41	16,32	-3,56	0,09	0,00001
DEFA1	NA	NA	15,80	12,29	12,14	11,77	-3,74	0,08	0,00015
FBN1	22,36	22,91	22,66	17,79	18,31	19,26	-4,19	0,05	0,00002

Supplementary Table S2: Oxidative stress-related proteins

Proteins	Functions
BDH2	3-hydroxybutyrate dehydrogenase type 2; Dehydrogenase that mediates the formation of 2,5-dihydroxybenzoic acid (2,5-DHBA), a siderophore that shares structural similarities with bacterial enterobactin and associates with LCN2, thereby playing a key role in iron assimilation and homeostasis. Plays a role in susceptibility to bacterial infection by providing an assimilable source of iron that is exploited by pathogenic bacteria (By similarity). Also acts as a 3-hydroxybutyrate dehydrogenase. (245 aa)
CPOX	Oxygen-dependent coproporphyrinogen-III oxidase, mitochondrial; Involved in the heme biosynthesis. Catalyzes the aerobic oxidative decarboxylation of propionate groups of rings A and B of coproporphyrinogen-III to yield the vinyl groups in protoporphyrinogen- IX. (454 aa)
DLD	Dihydrolipoyl dehydrogenase, mitochondrial; Lipoamide dehydrogenase is a component of the glycine cleavage system as well as an E3 component of three alpha-ketoacid dehydrogenase complexes (pyruvate-, alpha-ketoglutarate-, and branched- chain amino acid-dehydrogenase complex). The 2-oxoglutarate dehydrogenase complex is mainly active in the mitochondrion. A fraction of the 2-oxoglutarate dehydrogenase complex also localizes in the nucleus and is required for lysine succinylation of histones: associates with KAT2A on chromatin and provides succinyl-CoA to histone succinyltransferase. (509 aa)
GAPDH	Glyceraldehyde-3-phosphate dehydrogenase; Has both glyceraldehyde-3-phosphate dehydrogenase and nitrosylase activities, thereby playing a role in glycolysis and nuclear functions, respectively. Participates in nuclear events including transcription, RNA transport, DNA replication and apoptosis. Nuclear functions are probably due to the nitrosylase activity that mediates cysteine S-nitrosylation of nuclear target proteins such as SIRT1, HDAC2 and PRKDC. Modulates the organization and assembly of the cytoskeleton. (335 aa)
GPX3	Glutathione peroxidase 3; Protects cells and enzymes from oxidative damage, by catalyzing the reduction of hydrogen peroxide, lipid peroxides and organic hydroperoxide, by glutathione. (226 aa)
IMPDH1	Inosine-5'-monophosphate dehydrogenase 1; Catalyzes the conversion of inosine 5'-phosphate (IMP) to xanthosine 5'-phosphate (XMP), the first committed and rate-limiting step in the de novo synthesis of guanine nucleotides, and therefore plays an important role in the regulation of cell growth. Could also have a single-stranded nucleic acid-binding activity and could play a role in RNA and/or DNA metabolism. It may also have a role in the development of malignancy and the growth progression of some tumors; Belongs to the IMPDH/GMPR family. (599 aa)
MTHFD2	Bifunctional methylenetetrahydrofolate dehydrogenase/cyclohydrolase, mitochondrial; Although its dehydrogenase activity is NAD-specific, it can also utilize NADP at a reduced efficiency. Belongs to the tetrahydrofolate dehydrogenase/cyclohydrolase family. (350 aa)
P3H1	Prolyl 3-hydroxylase 1; Basement membrane-associated chondroitin sulfate proteoglycan (CSPG). Has prolyl 3-hydroxylase activity catalyzing the post- translational formation of 3-hydroxyproline in -Xaa-Pro-Gly- sequences in collagens, especially types IV and V. May be involved in the secretory pathway of cells. Has growth suppressive activity in fibroblasts. (804 aa)
P4HA1	Prolyl 4-hydroxylase subunit alpha-1; Catalyzes the post-translational formation of 4- hydroxyproline in -Xaa-Pro-Gly- sequences in collagens and other proteins; Belongs to the P4HA family. (534 aa)
P4HA2	Prolyl 4-hydroxylase subunit alpha-2; Catalyzes the post-translational formation of 4- hydroxyproline in -Xaa-Pro-Gly- sequences in collagens and other proteins. (535 aa)

P4HB	Protein disulfide-isomerase; This multifunctional protein catalyzes the formation, breakage and rearrangement of disulfide bonds. At the cell surface, seems to act as a reductase that cleaves disulfide bonds of proteins attached to the cell. May therefore cause structural modifications of exofacial proteins. Inside the cell, seems to form/rearrange disulfide bonds of nascent proteins. At high concentrations, functions as a chaperone that inhibits aggregation of misfolded proteins. At low concentrations, facilitates aggregation (anti-chaperone activity). (508 aa)
PLOD1	Procollagen-lysine,2-oxoglutarate 5-dioxygenase 1; Part of a complex composed of PLOD1, P3H3 and P3H4 that catalyzes hydroxylation of lysine residues in collagen alpha chains and is required for normal assembly and cross-linking of collagen fibrils (By similarity). Forms hydroxylysine residues in -Xaa-Lys- Gly- sequences in collagens. These hydroxylysines serve as sites of attachment for carbohydrate units and are essential for the stability of the intermolecular collagen cross-links (Probable). (727 aa)
PLOD3	Multifunctional procollagen lysine hydroxylase and glycosyltransferase LH3; Multifunctional enzyme that catalyzes a series of essential post-translational modifications on Lys residues in procollagen. Plays a redundant role in catalyzing the formation of hydroxylysine residues in -Xaa-Lys-Gly- sequences in collagens. Plays a redundant role in catalyzing the transfer of galactose onto hydroxylysine groups, giving rise to galactosyl 5-hydroxylysine. Has an essential role by catalyzing the subsequent transfer of glucose moieties. (738 aa)
PRDX3	Thioredoxin-dependent peroxide reductase, mitochondrial; Thiol-specific peroxidase that catalyzes the reduction of hydrogen peroxide and organic hydroperoxides to water and alcohols, respectively. Plays a role in cell protection against oxidative stress by detoxifying peroxides. Acts synergistically with MAP3K13 to regulate the activation of NF-kappa-B in the cytosol. (256 aa)
PXDN	Peroxidasin homolog; Displays low peroxidase activity and is likely to participate in H2O2 metabolism and peroxidative reactions in the cardiovascular system. Plays a role in extracellular matrix formation. (1479 aa)
SOD2	Superoxide dismutase [Mn], mitochondrial; Destroys superoxide anion radicals which are normally produced within the cells and which are toxic to biological systems. Belongs to the iron/manganese superoxide dismutase family. (222 aa)
SOD3	Extracellular superoxide dismutase [Cu-Zn]; Protect the extracellular space from toxic effect of reactive oxygen intermediates by converting superoxide radicals into hydrogen peroxide and oxygen. (240 aa)
SUMF1	Formylglycine-generating enzyme; Oxidase that catalyzes the conversion of cysteine to 3- oxoalanine on target proteins, using molecular oxygen and an unidentified reducing agent. 3- oxoalanine modification, which is also named formylglycine (fGly), occurs in the maturation of arylsulfatases and some alkaline phosphatases that use the hydrated form of 3-oxoalanine as a catalytic nucleophile. Known substrates include GALNS, ARSA, STS and ARSE. Belongs to the sulfatase-modifying factor family. (374 aa)

Supplementary Table S3: Angiogenesis-related proteins

Proteins	Functions
ADM	Proadrenomedullin N-20 terminal peptide; AM and PAMP are potent hypotensive and vasodilatator agents. Numerous actions have been reported most related to the physiologic control of fluid and electrolyte homeostasis. In the kidney, AM is diuretic and natriuretic, and both AM and PAMP inhibit aldosterone secretion by direct adrenal actions. In pituitary gland, both peptides at physiologically relevant doses inhibit basal ACTH secretion. Both peptides appear to act in brain and pituitary gland to facilitate the loss of plasma volume, actions which complement their hypotensive effects. (185 aa)
ANGPTL4	Angiopoietin-related protein 4; Mediates inactivation of the lipoprotein lipase LPL, and thereby plays a role in the regulation of triglyceride clearance from the blood serum and in lipid metabolism. May also play a role in regulating glucose homeostasis and insulin sensitivity (Probable). Inhibits proliferation, migration, and tubule formation of endothelial cells and reduces vascular leakage. Upon heterologous expression, inhibits the adhesion of endothelial cell to the extracellular matrix (ECM), and inhibits the reorganization of the actin cytoskeleton. (406 aa)
BMP4	Bone morphogenetic protein 4; Induces cartilage and bone formation. Also acts in mesoderm induction, tooth development, limb formation and fracture repair. Acts in concert with PTHLH/PTHRP to stimulate ductal outgrowth during embryonic mammary development and to inhibit hair follicle induction (By similarity). (408 aa)
CASP8	Caspase-8 subunit p10; Most upstream protease of the activation cascade of caspases responsible for the TNFRSF6/FAS mediated and TNFRSF1A induced cell death. Binding to the adapter molecule FADD recruits it to either receptor. The resulting aggregate called death-inducing signaling complex (DISC) performs CASP8 proteolytic activation. The active dimeric enzyme is then liberated from the DISC and free to activate downstream apoptotic proteases. Proteolytic fragments of the N-terminal propeptide (termed CAP3, CAP5 and CAP6) are likely retained in the DISC. Cleaves and activates CASP3. (538 aa)
CCBE1	Collagen and calcium-binding EGF domain-containing protein 1; Required for lymphangioblast budding and angiogenic sprouting from venous endothelium during embryogenesis. Belongs to the CCBE1 family. (406 aa)
CCN1	CCN family member 1; Promotes cell proliferation, chemotaxis, angiogenesis and cell adhesion. Appears to play a role in wound healing by up- regulating, in skin fibroblasts, the expression of a number of genes involved in angiogenesis, inflammation and matrix remodeling including VEGA-A, VEGA-C, MMP1, MMP3, TIMP1, uPA, PAI-1 and integrins alpha-3 and alpha-5. CCN1-mediated gene regulation is dependent on heparin-binding. Down-regulates the expression of alpha-1 and alpha-2 subunits of collagen type-1. Promotes cell adhesion and adhesive signaling through integrin alpha-6/beta-1. (381 aa)
CCN2	CCN family member 2; Major connective tissue mitoattractant secreted by vascular endothelial cells. Promotes proliferation and differentiation of chondrocytes. Mediates heparin- and divalent cation-dependent cell adhesion in many cell types including fibroblasts, myofibroblasts, endothelial and epithelial cells. Enhances fibroblast growth factor- induced DNA synthesis; Belongs to the CCN family. (349 aa)
CCN3	CCN family member 3; Immediate-early protein playing a role in various cellular processes including proliferation, adhesion, migration, differentiation and survival. Acts by binding to integrins or membrane receptors such as NOTCH1. Essential regulator of hematopoietic stem and progenitor cell function. Inhibits myogenic differentiation through the activation of Notch-signaling pathway. Inhibits vascular smooth muscle cells proliferation by increasing expression of cell-cycle regulators such as CDKN2B or CDKN1A independently of TGFB1 signaling. Ligand of integrins ITGAV:ITGB3 and ITGA5. (357 aa)

COL18A1	Collagen alpha-1(XVIII) chain; Probably plays a major role in determining the retinal structure as well as in the closure of the neural tube. Endostatin: Potently inhibits endothelial cell proliferation and angiogenesis. May inhibit angiogenesis by binding to the heparan sulfate proteoglycans involved in growth factor signaling (By similarity). Inhibits VEGFA-induced endothelial cell proliferation and migration. Seems to inhibit VEGFA-mediated signaling by blocking the interaction of VEGFA to its receptor KDR/VEGFR2. Modulates endothelial cell migration in an integrin-dependent manner. (1754 aa)
COL4A2	Collagen alpha-2(IV) chain; Type IV collagen is the major structural component of glomerular basement membranes (GBM), forming a 'chicken-wire' meshwork together with laminins, proteoglycans and entactin/nidogen. (1712 aa)
CTSH	Cathepsin H heavy chain; Important for the overall degradation of proteins in lysosomes; Belongs to the peptidase C1 family. (335 aa)
EFEMP2	EGF containing fibulin extracellular matrix protein 2; Belongs to the fibulin family. (443 aa)
EFNA1	Ephrin-A1, secreted form; Cell surface GPI-bound ligand for Eph receptors, a family of receptor tyrosine kinases which are crucial for migration, repulsion and adhesion during neuronal, vascular and epithelial development. Binds promiscuously Eph receptors residing on adjacent cells, leading to contact-dependent bidirectional signaling into neighboring cells. Plays an important role in angiogenesis and tumor neovascularization. The recruitment of VAV2, VAV3 and PI3-kinase p85 subunit by phosphorylated EPHA2 is critical for EFNA1-induced RAC1 GTPase activation and vascular endothelial cell migration. (205 aa)
ERAP1	Endoplasmic reticulum aminopeptidase 1; Aminopeptidase that plays a central role in peptide trimming, a step required for the generation of most HLA class I-binding peptides. Peptide trimming is essential to customize longer precursor peptides to fit them to the correct length required for presentation on MHC class I molecules. Strongly prefers substrates 9-16 residues long. Rapidly degrades 13-mer to a 9-mer and then stops. Preferentially hydrolyzes the residue Leu and peptides with a hydrophobic C-terminus, while it has weak activity toward peptides with charged C-terminus. (948 aa)
EXT1	Exostosin-1; Glycosyltransferase required for the biosynthesis of heparan- sulfate. The EXT1/EXT2 complex possesses substantially higher glycosyltransferase activity than EXT1 or EXT2 alone. Appears to be a tumor suppressor. Required for the exosomal release of SDCBP, CD63 and syndecan. (746 aa)
FGF2	Fibroblast growth factor 2; Acts as a ligand for FGFR1, FGFR2, FGFR3 and FGFR4. Also acts as an integrin ligand which is required for FGF2 signaling. Binds to integrin ITGAV:ITGB3. Plays an important role in the regulation of cell survival, cell division, cell differentiation and cell migration. Functions as a potent mitogen in vitro. Can induce angiogenesis. (288 aa)
GRN	Paragranulin; Secreted protein that acts as a key regulator of lysosomal function and as a growth factor involved in inflammation, wound healing and cell proliferation. Regulates protein trafficking to lysosomes and, also the activity of lysosomal enzymes. Facilitates also the acidification of lysosomes, causing degradation of mature CTSD by CTSB. In addition, functions as wound-related growth factor that acts directly on dermal fibroblasts and endothelial cells to promote division, migration and the formation of capillary-like tubule structures. (593 aa)
HSPG2	Basement membrane-specific heparan sulfate proteoglycan core protein; Integral component of basement membranes. Component of the glomerular basement membrane (GBM), responsible for the fixed negative electrostatic membrane charge, and which provides a barrier which is both size- and charge-selective. It serves as an attachment substrate for cells. Plays essential roles in vascularization. Critical for normal heart development and for regulating the vascular response to injury. Also required for avascular cartilage development. (4391 aa)

IGFBP5	Insulin-like growth factor-binding protein 5; IGF-binding proteins prolong the half-life of the IGFs and have been shown to either inhibit or stimulate the growth promoting effects of the IGFs on cell culture. They alter the interaction of IGFs with their cell surface receptors. (272 aa)
JAG1	Protein jagged-1; Ligand for multiple Notch receptors and involved in the mediation of Notch signaling. May be involved in cell-fate decisions during hematopoiesis. Seems to be involved in early and late stages of mammalian cardiovascular development. Inhibits myoblast differentiation (By similarity). Enhances fibroblast growth factor-induced angiogenesis (*in vitro*). (1218 aa)
LAMA5	Laminin subunit alpha-5; Binding to cells via a high affinity receptor, laminin is thought to mediate the attachment, migration and organization of cells into tissues during embryonic development by interacting with other extracellular matrix components. (3695 aa)
LIPA	Lysosomal acid lipase/cholesteryl ester hydrolase; Crucial for the intracellular hydrolysis of cholesteryl esters and triglycerides that have been internalized via receptor- mediated endocytosis of lipoprotein particles. Important in mediating the effect of LDL (low density lipoprotein) uptake on suppression of hydroxymethylglutaryl-CoA reductase and activation of endogenous cellular cholesteryl ester formation; Belongs to the AB hydrolase superfamily. Lipase family. (399 aa)
LTBP3	Latent-transforming growth factor beta-binding protein 3; Key regulator of transforming growth factor beta (TGFB1, TGFB2 and TGFB3) that controls TGF-beta activation by maintaining it in a latent state during storage in extracellular space. Associates specifically via disulfide bonds with the Latency-associated peptide (LAP), which is the regulatory chain of TGF-beta, and regulates integrin-dependent activation of TGF-beta. (1303 aa)
MAN1A2	Mannosyl-oligosaccharide 1,2-alpha-mannosidase IB; Involved in the maturation of Asn-linked oligosaccharides. Progressively trim alpha-1,2-linked mannose residues from Man(9)GlcNAc(2) to produce Man(5)GlcNAc(2); Belongs to the glycosyl hydrolase 47 family. (641 aa)
MDK	Midkine; Secreted protein that functions as cytokine and growth factor and mediates its signal through cell-surface proteoglycan and non- proteoglycan receptors. Binds cell-surface proteoglycan receptors via their chondroitin sulfate (CS) groups. Thereby regulates many processes like inflammatory response, cell proliferation, cell adhesion, cell growth, cell survival, tissue regeneration, cell differentiation and cell migration. Participates in inflammatory processes by exerting two different activities. Firstly, mediates neutrophils and macrophages recruitment to the sites of inflammation. (143 aa)
MEGF8	Multiple epidermal growth factor-like domains protein 8; Acts as a negative regulator of hedgehog signaling. (2845 aa)
MFGE8	Lactadherin short form; Plays an important role in the maintenance of intestinal epithelial homeostasis and the promotion of mucosal healing. Promotes VEGF-dependent neovascularization (By similarity). Contributes to phagocytic removal of apoptotic cells in many tissues. Specific ligand for the alpha-v/beta-3 and alpha-v/beta-5 receptors. Also binds to phosphatidylserine-enriched cell surfaces in a receptor-independent manner. Zona pellucida-binding protein which may play a role in gamete interaction. (387 aa)
MMP2	72 kDa type IV collagenase; Ubiquitinous metalloproteinase that is involved in diverse functions such as remodeling of the vasculature, angiogenesis, tissue repair, tumor invasion, inflammation, and atherosclerotic plaque rupture. As well as degrading extracellular matrix proteins, can also act on several nonmatrix proteins such as big endothelial 1 and beta- type CGRP promoting vasoconstriction. Also cleaves KISS at a Gly-|-Leu bond. Appears to have a role in myocardial cell death pathways. Contributes to myocardial oxidative stress by regulating the activity of GSK3beta. (660 aa)
NAXE	NAD(P)H-hydrate epimerase; Catalyzes the epimerization of the S- and R-forms of NAD(P)HX, a damaged form of NAD(P)H that is a result of enzymatic or heat-dependent hydration. This is a prerequisite for the S-specific NAD(P)H-hydrate dehydratase to allow the repair of both epimers of NAD(P)HX; Belongs to the NnrE/AIBP family. (288 aa)

PCSK5	Proprotein convertase subtilisin/kexin type 5; Serine endoprotease that processes various proproteins by cleavage at paired basic amino acids, recognizing the RXXX[KR]R consensus motif. Likely functions in the constitutive and regulated secretory pathways. Plays an essential role in pregnancy establishment by proteolytic activation of a number of important factors such as BMP2, CALD1 and alpha-integrins; Belongs to the peptidase S8 family. (1860 aa)
PDGFC	Platelet-derived growth factor C, receptor-binding form; Growth factor that plays an essential role in the regulation of embryonic development, cell proliferation, cell migration, survival and chemotaxis. Potent mitogen and chemoattractant for cells of mesenchymal origin. Required for normal skeleton formation during embryonic development, especially for normal development of the craniofacial skeleton and for normal development of the palate. Required for normal skin morphogenesis during embryonic development. Plays an important role in wound healing, (345 aa)
PLOD3	Multifunctional procollagen lysine hydroxylase and glycosyltransferase LH3; Multifunctional enzyme that catalyzes a series of essential post-translational modifications on Lys residues in procollagen. Plays a redundant role in catalyzing the formation of hydroxylysine residues in -Xaa-Lys-Gly- sequences in collagens. Plays a redundant role in catalyzing the transfer of galactose onto hydroxylysine groups, giving rise to galactosyl 5-hydroxylysine. Has an essential role by catalyzing the subsequent transfer of glucose moieties, giving rise to 1,2-glucosylgalactosyl-5-hydroxylysine residues. (738 aa)
PXDN	Peroxidasin homolog; Displays low peroxidase activity and is likely to participate in H(2)O(2) metabolism and peroxidative reactions in the cardiovascular system. Plays a role in extracellular matrix formation. (1479 aa)
RNF213	E3 ubiquitin-protein ligase RNF213; E3 ubiquitin-protein ligase involved in angiogenesis. Involved in the non-canonical Wnt signaling pathway in vascular development: acts by mediating ubiquitination and degradation of FLNA and NFATC2 downstream of RSPO3, leading to inhibit the non-canonical Wnt signaling pathway and promoting vessel regression. Also has ATPase activity. (5207 aa)
SCG2	Secretogranin-2; Neuroendocrine protein of the granin family that regulates the biogenesis of secretory granules. (617 aa)
SEMA3C	Semaphorin-3C; Binds to plexin family members and plays an important role in the regulation of developmental processes. Required for normal cardiovascular development during embryogenesis. Functions as attractant for growing axons, and thereby plays an important role in axon growth and axon guidance (By similarity). (751 aa)
SERPINE2	Glia-derived nexin; Serine protease inhibitor with activity toward thrombin, trypsin, and urokinase. Promotes neurite extension by inhibiting thrombin. Binds heparin; Belongs to the serpin family. (409 aa)
TGFBI	Transforming growth factor-beta-induced protein ig-h3; Plays a role in cell adhesion. May play a role in cell-collagen interactions (By similarity). (683 aa)
THBS1	Thrombospondin-1; Adhesive glycoprotein that mediates cell-to-cell and cell-to- matrix interactions. Binds heparin. May play a role in dentinogenesis and/or maintenance of dentin and dental pulp (By similarity). Ligand for CD36 mediating antiangiogenic properties. Plays a role in ER stress response, via its interaction with the activating transcription factor 6 alpha (ATF6) which produces adaptive ER stress response factors (By similarity). (1170 aa)
VEGFC	Vascular endothelial growth factor C; Growth factor active in angiogenesis, and endothelial cell growth, stimulating their proliferation and migration and also has effects on the permeability of blood vessels. May function in angiogenesis of the venous and lymphatic vascular systems during embryogenesis, and also in the maintenance of differentiated lymphatic endothelium in adults. Binds and activates KDR/VEGFR2 and FLT4/VEGFR3 receptors. (419 aa)

WNT5A Protein Wnt-5a; Ligand for members of the frizzled family of seven transmembrane receptors. Can activate or inhibit canonical Wnt signaling, depending on receptor context. In the presence of FZD4, activates beta-catenin signaling. In the presence of ROR2, inhibits the canonical Wnt pathway by promoting beta-catenin degradation through a GSK3-independent pathway which involves down-regulation of beta- catenin-induced reporter gene expression (By similarity). Suppression of the canonical pathway allows chondrogenesis to occur and inhibits tumor formation. Stimulates cell migration. (380 aa)
